# Hepatitis B virus DNA integration as a novel biomarker of hepatitis B virus-mediated pathogenetic properties and a barrier to the current strategies for hepatitis B virus cure

**DOI:** 10.3389/fmicb.2022.972687

**Published:** 2022-09-02

**Authors:** Romina Salpini, Stefano D’Anna, Livia Benedetti, Lorenzo Piermatteo, Upkar Gill, Valentina Svicher, Patrick T. F. Kennedy

**Affiliations:** ^1^Department of Experimental Medicine, University of Rome Tor Vergata, Roma, Italy; ^2^Barts Liver Centre, Barts and The London School of Medicine and Dentistry, Blizard Institute, Queen Mary University of London, London, United Kingdom; ^3^Department of Biology, University of Rome Tor Vergata, Roma, Italy

**Keywords:** HBV-DNA integration, HBV cure, HBV biomarkers, chronic HBV infection, hepatocellular carcinoma

## Abstract

Chronic infection with Hepatitis B Virus (HBV) is a major cause of liver-related morbidity and mortality worldwide. HBV-DNA integration into the human genome is recognized as a frequent event occurring during the early phases of HBV infection and characterizing the entire course of HBV natural history. The development of refined molecular biology technologies sheds new light on the functional implications of HBV-DNA integration into the human genome, including its role in the progression of HBV-related pathogenesis and in triggering the establishment of pro-oncogenic mechanisms, promoting the development of hepatocellular carcinoma. The present review provides an updated and comprehensive overview of the current body of knowledge on HBV-DNA integration, focusing on the molecular mechanisms underlying HBV-DNA integration and its occurrence throughout the different phases characterizing the natural history of HBV infection. Furthermore, here we discuss the main clinical implications of HBV integration as a biomarker of HBV-related pathogenesis, particularly in reference to hepatocarcinogenesis, and how integration may act as a barrier to the achievement of HBV cure with current and novel antiviral therapies. Overall, a more refined insight into the mechanisms and functionality of HBV integration is paramount, since it can potentially inform the design of *ad hoc* diagnostic tools with the ability to reveal HBV integration events perturbating relevant intracellular pathways and for identifying novel therapeutic strategies targeting alterations directly related to HBV integration.

## Introduction

Hepatitis B virus (HBV) is a major global health problem and a leading cause of death. According to recent WHO estimates, 270 million people have a chronic HBV infection, resulting in 800,000 deaths every year, attributed to cirrhosis and liver cancer (World Health Organization [WHO], 2017; Razavi-Shearer et al., 2018). In particular, hepatocellular carcinoma (HCC), which remains associated with a poor prognosis, is the fourth leading cause of cancer death worldwide (Sung et al., 2021). The lifetime risk of developing HCC is 10- to 100-fold greater for patients with chronic HBV infection than non-infected individuals, and this risk (although reduced) persists even with successful antiviral therapy and notably, also in patients with clinically resolved infection (El-Serag, 2012; Shi et al., 2012; Mak et al., 2020). In contrast with other aetiologies, a substantial number of HBV-infected individuals develop HCC without signs of liver damage, highlighting the existence of direct HBV pro-oncogenetic potential (Fattovich et al., 2004; Levrero and Zucman-Rossi, 2016). HBV-DNA integration in the hepatocytes’ genome is under intensive investigation for its role in promoting enhanced cell proliferation by chromosomal genome instability or producing chimeric viral-human RNAs/proteins with transactivating properties (Lau et al., 2014; Jin et al., 2019; Alvarez-Benayas et al., 2021). Furthermore, in the setting of HBeAg-negative chronic hepatitis B (CHB), HBV-DNA integration can represent a source for the production of HBsAg that can occur even when cccDNA is completely silenced, thus challenging the rationale for HBV functional cure, whose surrogate marker is HBsAg loss (Ringlander et al., 2020; Rydell et al., 2020; Meier et al., 2021). Considering this, the current review is dedicated to providing a comprehensive overview related to the issue of HBV-DNA integration during the course of HBV infection and its role in modulating HBV pathogenetic/oncogenetic properties. Novel insights on the methodological aspects for detecting HBV-DNA integrants and their potential role as an early biomarker for HCC development are also presented.

Overall, this review will assist in deciphering the current knowledge and identifying areas of future research to better understand the role of HBV-DNA integration in disease progression and the development of HCC.

## Replication cycle of hepatitis B virus and integration of viral DNA into the host genome

**FIGURE 1:**
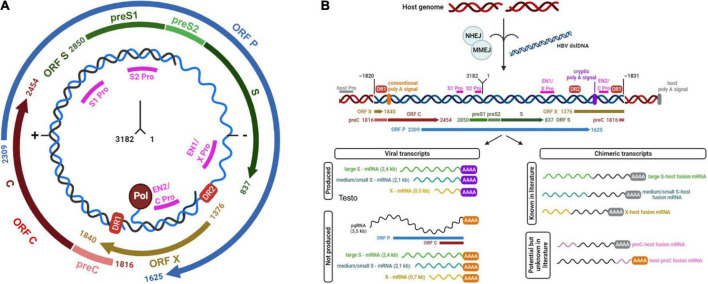
**(A)** Schematic representation of HBV genome. HBV genome is represented in relaxed circular form (rcDNA). The numbers at the center of the figure indicate the localization of first and last nucleotides. The four open reading frames or ORFs (S, P, X, C) are depicted. Pol circle indicates viral reverse transcriptase, linked to 3’-end of HBV-DNA. DR1 and DR2 red boxes indicate direct repeat regions 1 and 2: DR1 is present in both strands of HBV-rcDNA while DR2 is present only in the complete negative strand (−). Magenta lines show promoters (indicated as Pro) of different ORFs: in particular, enhancer 1 (EN1) acts as promoter of ORF X while enhancer 2 (EN2) acts as promoter for the expression of both ORF C and ORF P. **(B)** HBV double-strand linear DNA (dslDNA) integrated in host genome and its derived transcripts. The figure depicts the integration of HBV double-strand linear DNA (blue) into the host genome (red). The left-hand side of HBV-dslDNA is expected to be located at or near position 1820 of HBV genome, while the right-hand side at or near position 1831. These sites are not exclusive due to the integration of fragments of viral genome. Magenta lines show viral promoters (Pro), while gray line indicates a generic host promoter (host Pro). Viral open reading frames (ORFs) are depicted. DR1 and DR2 red boxes indicate viral direct repeat regions 1 and 2. Viral conventional poly A signal is showed as an orange bar (nucleotide positions: 1916–1921) while the cryptic poly A signal is represented by a violet bar (nucleotide positions: 1788–1793). The figure reports viral or viral-human transcripts produced from integrated dslDNA. The former derives from the stop of transcription at the site of the cryptic poly A signal, while the latter from the stop of transcription at a human poly A signal. In both panels, the numbering of nucleotides was based on the HBV DNA sequence with Genbank Accession #AB241115—genotype A.

Hepatitis B Virus (HBV) is a member of Hepadnaviridae family. It is an enveloped virus with a relaxed circular double-stranded DNA (dsDNA) genome of about 3.2 kbp. The genome is composed by four overlapping open reading frames (ORFs), encoding seven proteins. In particular, the ORF S encodes the three isoforms of HBV surface antigen (HBsAg), referred to as Large-, Middle-, and Small-HBsAg, the ORF C encodes the HBV capsid antigen (HBcAg) and the secreted HBV “e” antigen (HBeAg), the ORF P encodes the reverse transcriptase (RT) and lastly the ORF X the regulatory protein HBx with transactivating properties (Figure 1A; Liang, 2009; Urban et al., 2010; Tu et al., 2017).

Hepatitis B virus replication cycle starts with attachment and entry into hepatocytes through low specificity interactions between HBsAg and heparan sulfate proteoglycans on the surface of hepatocytes and then through highly specific interaction between viral pre-S1 domain of HBsAg and cellular sodium taurocholate co-transporting polypeptide (NTCP) (Beck and Nassal, 2007; Liang, 2009; Urban et al., 2010; Tu et al., 2017).

After entry, the nucleocapsid is released into the cytoplasm and reaches the nucleus, where the HBV relaxed circular DNA (rcDNA) is converted into the so-called covalently closed circular DNA (cccDNA). This process is mediated by the nuclear host cell components of DNA repair machinery and leads to the synthesis of the episomal template used for the transcription of both forms of HBV messenger RNAs (mRNAs): subgenomic mRNAs and pregenomic RNA (pgRNA) (Urban et al., 2010; Tu et al., 2017). The former contain the information to produce the three HBsAg isoforms and the HBx protein while the latter is mainly used as a template for the synthesis of HBV-DNA and for the translation of proteins such as the RT, HBcAg, and HBeAg. All the viral mRNA molecules share the same 3′ terminus and are polyadenylated near position 1931, using the conventional polyA signal located at positions 1916–1921 (Liang, 2009; Urban et al., 2010; Tu et al., 2017).

Reverse transcription occurs within the newly synthetized nucleocapsids where the pgRNA and the polymerase are translocated and so the new genome is produced inside the viral progeny (Beck and Nassal, 2007; Urban et al., 2010; Tu et al., 2017). This mechanism of replication makes HBV a particularly interesting virus, as its genome is constituted by DNA, it still requires an RNA-intermediate for genome replication.

Most nucleocapsids, produced by HBV-infected cells, contain relaxed circular DNA. These can be enveloped and secreted as virions or migrate to the nucleus and re-constitute the intranuclear cccDNA pool. Conversely, a small proportion of nucleocapsids contains double-stranded linear DNA (dslDNA), that can be released as enveloped virions or can be transported to the nucleus contributing to the further replenishment of the cccDNA pool *via* homologous recombination (Tu et al., 2017).

Notably, intranuclear HBV-dslDNA can also integrate into the host cell genome (Tu et al., 2017). HBV-DNA integrations occur at the site of cellular double-stranded DNA breaks by exploiting cellular repair mechanisms such as non-homologous or microhomology mediated end-joining (NHEJ and MMEJ) (Bill and Summers, 2004; Tu et al., 2018; Figure 1B). The left-hand side of HBV-dslDNA is expected to be located at or near position 1820 of HBV genome, while the right-hand side is expected to be located at or near position 1831 (Mason et al., 2016; Figure 1B). However, these positions can vary since the error-prone host DNA repair pathways can introduce terminal truncations during the process of integration (Freitas et al., 2018).

Due to this organization of dslDNA, integrated HBV-DNA cannot support the synthesis of pgRNA, HBcAg, and RT, and thus it cannot represent a source for the production of new viral particles (Figure 1B; Tu et al., 2017). Conversely, in integrated HBV-DNA, the promoters of ORF S are intact and functional, thus allowing the synthesis of mRNAs for the L-, M-, and S-HBsAg (Shamay et al., 2001; Tu et al., 2017; Figure 1B). However, in integrated HBV-DNA, the conventional poly-A signal is located upstream of the promoters of ORF S (positions 1916–1921) and thus cannot be used (Figure 1B). For this reason, the transcription of ORF S can be terminated at a recently identified viral cryptic poly A signal (located at positions 1788–1793) (Freitas et al., 2018) or can pass the virus-host junction and continue into the host sequences until a host poly A signal is reached (Figure 1B). This process can give origin to chimeric virus-host mRNAs (Schutz et al., 1996; Kairat et al., 1999; Freitas et al., 2018; Figure 1B). Very limited information is known about the viral cryptic polyA signal and its activation during the transcription of dslDNA (Kairat et al., 1999).

Similarly, the promoter of the ORF X is functional in integrated HBV-DNA and can induce the production of C-terminal truncated HBx proteins that can retain their transactivation properties (Kumar et al., 1996; Tu et al., 2017; Figure 1B). Furthermore, there is evidence for an un-interrupted transcription by the cellular RNA-polymerase that can favor the production of chimeric HBx-host transcripts (Tu et al., 2017; Ruan et al., 2019; Figure 1B). Both truncated HBx forms and chimeric transcripts are currently being studied for their role in HBV-driven hepatocarcinogensis (Sung et al., 2012; Tu et al., 2017; Ruan et al., 2019).

Beyond HBV- dsl DNA, a recent study has also shown the integration of fragments of viral genome that could give origin to the production of truncated HBV proteins (Li et al., 2022).

The understanding of the above-mentioned mechanisms, underlying the process of HBV-DNA integration, has required several efforts and in particular a constant improvement of molecular techniques with the ability to properly identify and to evaluate the localization of HBV-DNA integration in the human genome. Since the first studies in the 1980s [based on Southern blotting, *in situ* hybridization (ISH) and Polymerase Chain Reaction (PCR)] significant progress has been made. In particular, the recent introduction of innovative next-generation sequencing technologies has allowed to sequence the entire human genome, providing a strong and rapid enlargement in the current knowledge regarding the occurrence of HBV integration, its localization, as well as its functional impact on the human genome (cite paragraph 8 for an overview of the main techniques utilized for revealing HBV-DNA integration).

## HBV-DNA integration during the natural history of hepatitis B virus infection

HBV-DNA integration has been shown to be present from the very early stages of HBV infection. It is observed not only in HCC and cirrhotic patients with chronic hepatitis (Brechot et al., 1980; Shafritz et al., 1981; Mason et al., 2010), but also in patients with acute HBV infection (Kimbi et al., 2005). These observations are consistent with the results obtained in woodchuck and duck animal models with hepatitis B. Indeed, viral DNA integration into the host cellular genome is a common characteristic of the Hepadnaviridae family (Tu et al., 2017).

### HBV-DNA integration in the setting of acute hepatitis B virus infection

There is a paucity of studies evaluating HBV-DNA integration in acute infections, mainly due to ethical issues in sampling liver tissue in this setting (Pollicino and Caminiti, 2021). Previous studies in patients developing fulminant hepatitis, have shown that HBV-DNA integration can occur in the first weeks of infection and involve multiple sites of the host genome (Scotto et al., 1983; Lugassy et al., 1987), in line with what is observed in animal and *in vitro* models (Yang and Summers, 1999; Summers and Mason, 2004; Tu et al., 2018).

### Evaluation of viral integration events in HBeAg-positive phases of chronic infection

The natural history of chronic HBV infection has been categorized in different phases (Lampertico et al., 2017; Pollicino and Caminiti, 2021) on the basis of specific biochemical, serological, and virological characteristics, including HBeAg status, serum HBV-DNA, and alanine aminotransferase (ALT) levels.

The first phase, defined as “HBeAg-positive chronic infection,” is characterized by high levels of HBV-DNA (∼ 10^10^ virions per mL), reflecting high rates of viral replication, while ALT levels remain normal despite the presence of an HBV-specific-T-cell response (Bertoletti and Kennedy, 2015; Gish et al., 2015; Mason et al., 2016; Park et al., 2017). This phase is then followed by HBeAg-positive chronic hepatitis, characterized by elevated ALT and reduction in serum HBV-DNA, reflecting the activation of an antiviral immune response that can progressively constrain viral replication (Pollicino and Caminiti, 2021). The establishment of a vigorous host immune response can promote necroinflammation, accelerating the progression toward cirrhosis (Pollicino and Caminiti, 2021).

**FIGURE 2:**
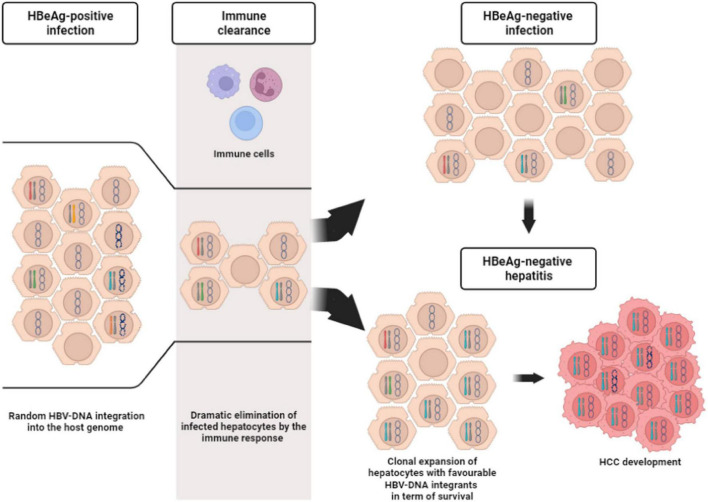
Description of the course of hepatocytes during chronic HBV infection. HBeAg-positive infection is characterized by a huge number of infected hepatocytes, as a consequence of high viral replication levels. The infected hepatocytes can harbor only cccDNA inside the nucleus (represented by the circular molecule) or also HBV-DNA randomly integrated into the host genome (represented by differently colored chromosomes). Not infected hepatocytes are indicated by cells with empty nucleus. Immune responses (depicted by the three different cells at the top of the gray stripe) reduce the number of the infected hepatocytes. From this stage the infection can run into two different outcomes. The former is represented by HBeAg-negative infection, where the pool of infected hepatocytes (with/without HBV-DNA integrants) remains limited, while the latter is represented HBeAg-negative hepatitis. In this stage, the immune response can induce the clonal selection and expansion of infected hepatocytes with favorable HBV-DNA integrants in term of survival. This process can hesitate in HCC development.

Recent studies have focused on the issue of HBV-DNA integration in the setting of HBeAg-positive phases of chronic HBV infection (Mason et al., 2016; Budzinska et al., 2018b; Rydell et al., 2020). Notably, we have previously demonstrated the presence of HBV-DNA integration events in all patients with HBeAg-positive chronic infection and hepatitis (Mason et al., 2016). In particular, by using an inverse PCR approach, we identified 500 unique HBV-DNA integrants; 246 of which were randomly located in transcribed regions (231 map to introns and 13 to exons, 1 at an intron/exon boundary) (Mason et al., 2016). These results have been confirmed in a recent metanalysis showing a higher frequency of HBV-DNA integrants in HBeAg-positive than HBeAg-negative patients (Budzinska et al., 2018a). It is plausible that the high levels of HBV replication, characterizing HBeAg-positive phases, can promote the abundant production of HBV-DNA intermediates that can undergo the integration process, posing the basis for the initiation of mechanisms underlying HBV oncogenic potential (Figure 2). This concept challenges the notion of HBeAg-positive infection as a quiescent disease phase (Bertoletti and Kennedy, 2015), raising the question about early treatment initiation in this subset of patients.

### Evaluation of viral integration events in HBeAg-negative phases of chronic infection

During the HBeAg-positive chronic hepatitis phase, the activation of an efficient immune response against HBV can lead to a progressive decline of HBeAg and serum HBV-DNA, reflecting a decrease in the burden and/or transcriptional activity of cccDNA (Kennedy et al., 2017; Pollicino and Caminiti, 2021). This determines the entry into the “HBeAg-negative chronic infection” phase, which is characterized by HBeAg-negativity, low serum HBV-DNA (usually < 2,000 IU/ml) and normal serum ALT (Pollicino and Caminiti, 2021). As this immune-control state is maintained, patients have a low risk of liver disease progression (Lampertico et al., 2017). Nevertheless, approximately one third of patients lose this “immune control” and progress to the “HBeAg-negative chronic hepatitis” phase (Lampertico et al., 2017; Pollicino and Caminiti, 2021). This disease phase is characterized by fluctuating or increasing serum HBV-DNA followed by elevations in serum ALT that can exacerbate liver damage thus accelerating the progression toward cirrhosis and HCC (Raimondo et al., 1990; Hsu et al., 2002). This phase is characterized by HBeAg-negativity due to the emergence of specific mutations in the pre-core and/or basal core promoter regions of the HBV genome that abolish or downregulate HBeAg production (Laras et al., 1998; Revill et al., 2020).

**FIGURE 3:**
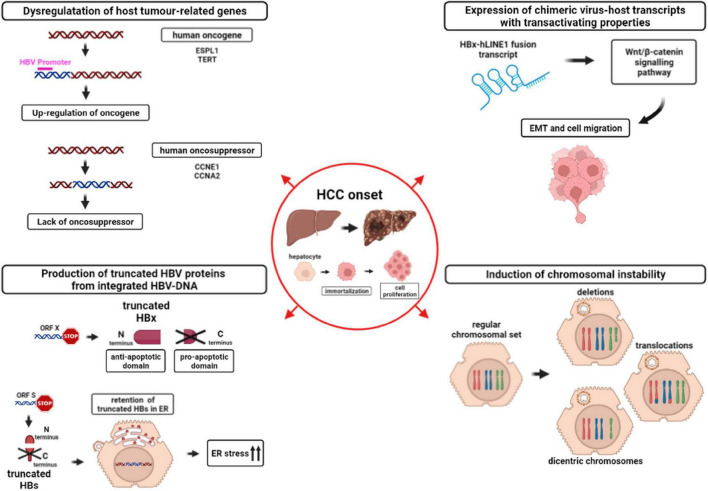
Mechanisms involved in HCC onset caused by HBV-DNA integration into the host genome. The figure depicts the downstream effects of HBV-DNA integration favoring HCC onset: (i) *Dysregulation of host tumor-related genes (top-left)* due to the up-regulation of host oncogenes (red) or to the lack of oncosuppressors. Magenta line indicates a generic HBV promoter; (ii) *Expression of chimeric virus-host transcripts with transactivating properties (top-right).* HBx-human long interspersed nuclear element 1 (hLINE1) fusion transcript, derived from integrated HBV-DNA, can activate the Wnt/β-catenin signaling pathway, leading to epithelial-mesenchymal transition (EMT) and cell migration; (iii) *Production of truncated HBV proteins from integrated HBV-DNA (bottom-left)*. HBV ORF X integration into the host genome can lead to the synthesis of a truncated HBx protein, retaining the anti-apoptotic N-terminal domain and lacking the pro-apoptotic C-terminal domain. Similarly, HBV ORF S integration can lead to the synthesis of truncated HBs proteins that are retained in the membrane of endoplasmic reticulum (ER), triggering ER stress, and in turn activating pro-oncogenic pathways; iv) *Induction of chromosomal instability (bottom-right).* The hepatocyte with a regular chromosomal set can develop chromosomal aberrations, such as deletions, translocations or dicentric chromosomes, as a consequence of HBV-DNA integration into the genome.

A lower rate of HBV-DNA integration has been observed in HBeAg-negative than HBeAg-positive patients, presumably reflecting a more limited pool of infected hepatocytes (Budzinska et al., 2018a; Rydell et al., 2020; Figure 2). Indeed, it has been hypothesized that, during the immune clearance phase and HBeAg seroconversion, the development of a strong immune response may favor the selection of those hepatocytes in which HBV-DNA integrations have conferred a selective advantage in terms of survival and escape from cytotoxic immune response (Budzinska et al., 2018a; Figure 2). Furthermore, this survival advantage is a key event since it can promote the progressive accumulation of chromosomal aberrations paving the way to the neoplastic transformation of the hepatocytes and in turn HCC development (Tu et al., 2017; Svicher et al., 2021; Figure 3).

We have recently addressed the issue of HBV-DNA integration in the setting of HBeAg-negativity (Svicher et al., 2021). By analyzing the whole exome of hepatocytes, this study has shown that HBV-DNA integration occurs not only in a notable proportion of patients with high HBV-DNA levels (55.6%), but also at significant frequency in patients with low (25%) and moderate viremia (14.3%), despite a more limited HBV reservoir (Svicher et al., 2021). Furthermore, by applying ddPCR, we found that HBV-DNA integrants occurred with a prevalence ranging from 0.5 up to 158 events per 1,000 hepatocytes, potentially suggesting a potential clonal expansion of hepatocytes harboring these HBV-DNA integrations (Svicher et al., 2021). This is supported by gene ontology analysis revealing the localization of HBV-DNA integration in human genes (NUP85, ANKRD52, ELAC2, and AGBL5) known to be involved in the regulation of cell proliferation and also in promoting the neoplastic transformation of different cell types including hepatocytes (Noda et al., 2006; Yu et al., 2017). Interestingly, the rate of HBV-DNA integration varies according to HBV genotypes, with genotype D characterized by the highest prevalence (38.9%) followed by genotype E (33.3%) and genotype C (22.2%) (Svicher et al., 2021). No results were available for the other genotypes due to very limited samples size.

Furthermore, the evidence of HBV-DNA integration in HBeAg negative low viremic patients with a limited HBV reservoir can be a reminder that even patients, not meeting treatment criteria, remain at risk of disease progression, supporting a re-evaluation of treatment candidacy (Svicher et al., 2021). This is in line with a recent study showing that treatment with NUCs can reduce the number of transcriptionally active HBV integrations, suggesting that this NUC-mediated effect should be considered in patients’ management (Hsu et al., 2022).

### HBV-DNA integration in the setting of occult HBV infection

Occult HBV infection (OBI) is defined as the presence of replication-competent HBV-DNA in the liver and/or in the blood of HBsAg-negative individuals (Raimondo et al., 2019). In particular, OBI is characterized by the long-lasting persistence of cccDNA in hepatocytes, whose transcriptional activity is strongly suppressed by the host’s defense mechanisms (Raimondo et al., 2019).

Based on the HBV-specific antibody profiles, OBI is mainly characterized by positivity to hepatitis B core antibody (anti-HBc) with or without hepatitis B surface anti-body (anti-HBs) (Raimondo et al., 2019).

Occult HBV infection can occur either following the resolution of acute hepatitis B or after decades of chronic HBV infection. Although the risk of disease progression is particularly low, there is evidence that HBV-DNA integration can occur also in the setting of OBI and can contribute to HBV-mediated carcinogenesis (Saitta et al., 2015; Chen et al., 2019). In particular, a previous study showed that the prevalence of HBV-DNA integrations in hepatic tumor tissues from OBI patients is quite high and can involve regulatory and functional host genes modulating cell proliferation (Saitta et al., 2015), highlighting a potential oncogenic risk also in this phase.

## HBV-DNA integration as a biomarker in mediating hepatitis B virus-related tumorigenesis

HBV-DNA integration is known to result in the dysregulation of genes in the neighborhood of the insertion site (Bailey and Murnane, 2006; Feitelson and Lee, 2007; Péneau et al., 2022; Ramirez et al., 2021; Álvarez et al., 2021). In the setting of hepatocarcinogenesis, the insertional mutagenesis of HBV-DNA can enhance expression of oncogenes, inactivate oncosuppressor expression, generate chimeric or truncated transcripts. Furthermore, HBV-DNA integration can also promote genome instability leading to the accumulation of chromosomal aberrations even at long distance from the integration site (Bailey and Murnane, 2006; Feitelson and Lee, 2007; Zhao et al., 2016; Yu et al., 2017; Álvarez et al., 2021; Bousali et al., 2021; Péneau et al., 2022; Ramirez et al., 2021). These mechanisms can trigger the clonal selection of hepatocytes with enhanced survival and proliferative properties, causing their neoplastic transformation (Ding et al., 2012; Fujimoto et al., 2012; Zhao et al., 2016; Bousali et al., 2021; Lin et al., 2021).

Hereafter, we discuss in more details the downstream effects of HBV-DNA integration in (i) dysregulating host tumor-related genes, (ii) inducing the expression of chimeric virus-host transcripts with transactivating properties, and (iii) producing truncated HBV proteins, and (iv) causing chromosomal instability (Figure 3).

### Dysregulation of host tumor-related genes

HBV-DNA integration can target directly genes regulating crucial intracellular pathways as cell cycle regulation, cell immortalization, cell-to-cell interaction, and cell signaling, thus inducing their altered gene expression, a process that can mediate the acquisition of pro-oncogenic properties by the involved hepatocytes (Paterlini-Bréchot et al., 2003; Murakami et al., 2005; Saigo et al., 2008; Sung et al., 2012; Kawai-Kitahata et al., 2016; Figure 3).

Despite the fact that HBV-DNA integration during chronic HBV infection is found to be randomly distributed across the host genome in non-tumor tissues (Budzinska et al., 2018b; Péneau et al., 2022), a peculiar recurrence of HBV-DNA integrations at the level of specific genes involved in carcinogenic pathways has been revealed in studies analyzing tumor liver samples. Among them, the most frequent targets of HBV-DNA integration found to be associated with HCC are represented by: TERT (telomerase reverse transcriptase), MAPK1 (mitogen-activated protein kinase 1), MLL2 and MLL4 (myeloid/lymphoid or mixed-lineage leukemia 2 and 4), CCNE1 and CCNA2 (cyclin E1 and A2), TP53 (tumor protein p53), CTNNB1 (Catenin Beta 1) FAR2 (fatty acyl-coA reductase 2), ITPR1 (inositol 1,4,5-trisphosphate receptor type 1), and IRAK2 (interleukin 1 receptor associated kinase 2) (Paterlini-Bréchot et al., 2003; Murakami et al., 2005; Saigo et al., 2008; Sung et al., 2012; Kawai-Kitahata et al., 2016). Among them, the enrichment of HBV-DNA integration in TERT, CCNE1, and CCNA2 genes in tumoral samples has also been confirmed by analyzing the publicly available database VISDB, collecting 20558 HBV-DNA integration sites in tumor/peritumor/non-tumor liver samples from 45 publications (Tang et al., 2020). In particular, ≥90% of HBV-DNA integration occurring in TERT, CCNE1, or CCNA2 is revealed in liver tumor samples compared to ≤10% in non-tumoral ones reported in VISDB, corroborating their role in mechanisms underlying HBV-driven carcinogenesis.

HBV-DNA integration in the TERT gene is considered the most important hotspot of HBV integration in tumor samples (Sze et al., 2021) and has been associated with a more aggressive tumor behavior and a significantly poorer survival (Zhao et al., 2016; Cui et al., 2020). In particular, the integration of the HBV enhancer 1 upstream of the gene encoding TERT has been recently shown to upregulate TERT expression (Péneau et al., 2022), resulting in its increased capability to maintain telomere integrity and stability of damaged hepatocytes. This is a critical event in permitting hepatocytes to overcome the mechanisms of cell senescence and, in turn, driving neoplastic transformation (Jang et al., 2021; Sze et al., 2021).

### Expression of chimeric virus-host transcripts with transactivating properties

As previously mentioned, the un-interrupted transcription by the cellular RNA-polymerase can lead to the production of fusion HBV-human transcripts (Figure 3).

The most frequently described HBV-human fusion transcripts are those containing HBx linked to the “long interspersed nuclear element 1” (LINE1), that is usually silent in the hepatocytes (Heikenwalder and Protzer, 2014; Lau et al., 2014; Liu et al., 2021). The integration of ORF X upstream human LINE1 (hLINE1) can induce the expression of a fusion HBx-hLINE1 transcript, whose detection was correlated with a shorter patients’ survival (Heikenwalder and Protzer, 2014; Lau et al., 2014; Zhang Y. et al., 2021). This fusion HBx-hLINE1 transcript is suggested to act as a long non-coding RNA, capable of activating the Wnt/β-catenin signaling pathway and to influence the epithelial-mesenchymal transition and in turn to enhance cell migration (Heikenwalder and Protzer, 2014; Lau et al., 2014; Zhang Y. et al., 2021).

### Production of truncated hepatitis B virus proteins from integrated HBV-DNA

HBV integration can also promote the production of viral proteins (both HBx and HBs) and/or their altered forms, which may contribute to viral persistence, to continuous liver damage and ultimately to HCC development (Shamay et al., 2001; Tu et al., 2001; Wang et al., 2003, 2006, 2010; Ning-Fang et al., 2008; Warner and Locarnini, 2008; Sze et al., 2013; Ng et al., 2016; Salpini et al., 2017; Zhang Y. et al., 2021; Figure 3).

Indeed, as previously mentioned, as a result of HBV integration, the promoter of the ORF X is functional in integrated HBV-DNA and can induce the production of HBx proteins, lacking the C-terminal domain known to have pro-apoptotic properties (Shamay et al., 2001; Zhang Y. et al., 2021; Figure 3). The resulting overexpression of the C-terminal truncated HBx can inhibit the apoptosis and induce the acquisition of stem cell-like properties, thus promoting the neoplastic transformation of the hepatocytes (Tu et al., 2001; Ning-Fang et al., 2008; Wang et al., 2010; Sze et al., 2013; Ng et al., 2016).

Similarly, recent studies have highlighted that the ORF S can be frequently involved in HBV-DNA integration events in liver tumor tissues (Hu et al., 2020; Jang et al., 2020). In particular, it has been observed that the integration of fragments of viral genome can lead to the production of truncated HBsAg, lacking the C-terminal domain. These C-terminal truncated HBsAg forms cannot be properly secreted, but are retained in the membrane of the endoplasmic reticulum, thus activating intracellular signaling known to promote cell proliferation and to represent a factor contributing to HCC onset (Wang et al., 2003, 2006; Warner and Locarnini, 2008; Salpini et al., 2017; Figure 3). Furthermore, the accumulation of these truncated forms of HBsAg in the endoplasmic reticulum can cause cellular oxidative stress, known to augment DNA damage with consequent double-stranded breaks in which dsl HBV-DNA can be integrated. Interestingly, it has also been demonstrated that these aberrant HBsAg forms may inhibit DNA double-stranded break repair thus contributing genomic instability (Hsieh et al., 2015) further promoting the neoplastic transformation of hepatocytes (Wang et al., 2006; Hsieh et al., 2015).

### Induction of chromosomal instability

Lastly, there is increasing evidence showing that HBV-DNA integration in the human genome can promote a status of generalized genomic instability, that increases the risk of accumulating genomic rearrangements, further promoting the neoplastic transformation of hepatocytes (Ding et al., 2012; Zhao et al., 2016; Tu et al., 2017; Figure 3).

Recently, by analyzing HBV-DNA integration profiles of 296 liver tumor samples, a recent study unraveled that the insertion of HBV-DNA into the human genome can cause dramatic genetic aberrations, including non-homologous chromosomal fusions, dicentric chromosomes and long telomeric deletions, that may also lead to loss of tumor suppressor genes (such as TP53, ARID1A, RB1, RPS6KA3, and IRF2) (Álvarez et al., 2021; Figure 3). Furthermore, by applying *ad hoc* mathematical models, these genomic rearrangements have been estimated to occur several years (up to 20 years) before cancer diagnosis, supporting their crucial role as early drivers of hepatocarcinogenesis (Álvarez et al., 2021). In a similar direction, recent studies showed that HBV-DNA integration can profoundly modify the architecture of human genome by altering cancer-related genes even at long distance from integration site (Péneau et al., 2022; Ramirez et al., 2021; Li et al., 2022).

## Hepatitis B virus integration as a diagnostic biomarker of hepatocellular carcinoma occurrence and recurrence

The increasing data on the role of HBV-DNA integration in promoting pro-oncogenic mechanisms, together with the relevant advancement in next-generation sequencing technologies, have raised growing interest in the possibility to detect HBV-DNA integration as a potential novel prognostic marker for HCC.

A recent metanalysis, including >18,000 HBV-DNA integration sites from tumor samples, identified a total of 396 recurrently targeted genes, of which 28 recurred in at least 10 HCC patients (Lin et al., 2021). This metanalysis is particularly relevant since it paves the way for the identification of key cellular genes, representing hot spots of HBV-DNA integration associated with HCC onset. The ultimate goal of these studies will be the generation of diagnostic gene panels, based on refined next generation sequencing methods, that could reveal the presence of HBV-DNA integrations involving those genes associated with oncogenesis, potentially acting as an early biomarker for HCC development (Table 1).

**TABLE 1:** Diagnostic application of HBV-DNA integration as a novel biomarker for HCC occurrence and recurrence.

Main findings	Potential application of HBV DNA integration in HCC diagnosis	References
Specific cellular genes involved in tumorigenic pathways have been recognized as hot spots of HBV integration in patients with HCC	Development of diagnostic gene panels, based on refined next-generation sequencing methods, that could reveal, in liver biopsies or in blood, the presence of HBV integrations involving those genes associated with oncogenesis, thus potentially acting as an early biomarker of HCC onset	Zhao et al., 2016; Li et al., 2018; Hu et al., 2020; Lin et al., 2021; Zheng et al., 2021
Chimeric HBV-human DNA resulting from HBV integration in genes involved in tumorigenic pathways can persist despite resection and anticipate HCC recurrence	Development of molecular assays for detecting and quantifying circulating HBV-human chimeric DNAs in blood, deriving from HBV integrations already recognized in primary HCC, as non-invasive biomarker of HCC recurrence	Li C. L. et al., 2020

Promising data have also emerged on the utility of circulating HBV-human chimeric DNA, resulting from HBV-DNA integration events, as a useful non-invasive biomarker for early identification of HCC development and its recurrence (Li et al., 2018; Liu et al., 2021; Zhang D. et al., 2021; Table 1). In 2019, a study demonstrated, for the first time, the presence of cell-free chimeric HBV-human DNA from blood samples of 20 patients with chronic HBV infection. These chimeric DNAs derived from 87 different HBV integration sites and were particularly enriched in tumor-related genes, thus suggesting, the possibility to use chimeric human-HBV DNA in blood as circulating biomarker for HCC (Li et al., 2018). In 2021, by applying a novel approach of Circulating Single-Molecule Amplification and Resequencing, another study confirmed that most recurrent integration events detected in blood cell-free DNA have originated from tumor tissues, corroborating the potential utility of non-invasive detection of HBV-DNA integration as a circulating tumor marker for HBV-related HCC (Zheng et al., 2021).

In keeping with these findings, a recent study detected HBV-human DNA in 97.7% of patients with HBV-related HCC (Li C. L. et al., 2020). Notably, by analyzing cell-free DNA from blood samples 2 months following HCC resection, the same HBV-human chimeric DNA were also found in 10 cases (23.3%), nine of whom (90%) experienced HCC recurrence within a year (Li C. L. et al., 2020). These data demonstrate that chimeric DNA resulting from HBV-DNA integration can persist despite resection and can also represent a potential novel tool for early detection of HCC recurrence.

## Hepatitis B virus integration in lymphoid cells and potential role in lymphomagenesis

Growing evidence highlights that an alternative active HBV reservoir is represented by immune cells, providing relevant sites for HBV persistence. Specifically, productive HBV infection has been demonstrated to occur in hematopoietic stem cells (HSCs) from HBV chronically infected patients (Romet-Lemonne et al., 1983; Elfassi et al., 1984; Ma et al., 2012) as well as *in vitro* experiments (Zeldis et al., 1986; Steinberg et al., 1990). Furthermore, HBV-DNA and its replicative intermediates (cccDNA, HBV-RNAs) were also found in circulating peripheral blood mononuclear cells (PBMCs), as well as singularly in B and T cells subsets, monocytes and NK cells from patients with acute or chronic HBV infection (Stoll-Becker et al., 1997; Trippler et al., 1999; Lee et al., 2015).

Moreover, peripheral lymphoid cells have also been recognized as an active site of HBV-DNA integration, detected with high frequency in all phases of HBV infection, including acute and occult hepatitis B (Pontisso et al., 1984; Laure et al., 1985; Pasquinelli et al., 1986; Table 2).

**TABLE 2:** Current knowledge on HBV integration in blood cells.

Findings on HBV-DNA integration in blood cell	Study population	Frequency of HBV integration in blood cells	Technique for detecting HBV integration	References
First evidence of HBV-DNA integration in PBMCs of HBV-infected patients	16 CHB patients	PBMCs (25%)	Southern blot	Pontisso et al., 1984
	4 HIV + patients (1 CHB and 3 OBI)	PBMCs (100%)	Southern blot	Laure et al., 1985
HBV-DNA integration in PBMCs occurs in all phases of HBV infection, including acute and occult infection	38 HBsAg + patients (8 AHB; 21 CHB, 6 CI, 3 HCC) and 34 OBI patients	PBMCs (100% AHB, 85.7% CHB; 33.3%; 66.6% HCC and 35.2% OBI)	Southern blot	Pasquinelli et al., 1986
First sequencing analysis of HBV-human junctions in the setting of HBV-DNA integration of PBMCs	10 CHB patients	PBMCs (20%)	Nested-PCR	Laskus et al., 1999
Accumulating evidences on HBV-DNA integration as a frequent event at level of PBMCs of patients with ongoing or past HBV infection	7 CHB patients; 9 OBI	PBMCs (42.8% CHB; 22.2% OBI)	Alu-PCR	Murakami et al., 2004
	21 OBI	PBMCs (14.2%)	Inverse-PCR	Umeda et al., 2005
	30 CHB adults and 19 CHB children	PBMCs (86% adults; 65% children)	Sanger Sequencing	Wang et al., 2008
Multiple HBV-DNA integration events revealed in PBMCs of CHB patients with non-Hodgkin lymphoma (NHL)	12 CHB with NHL diagnosis	Lymphoid malignant tissues (50%)	Next generation sequencing	Li M. et al., 2020
HBV-DNA integration in PBMCs is enriched at level of genes associated with tumorigenesis. Patterns of HBV integration are sheared by tumoral liver cells and PBMCs.	42 CHB patients	PBMCs (81%) A total of 271 integration events of which 58 in coding genes involved in oncogenetic pathway	Alu-PCR; Next-generation sequencing	Lau et al., 2020
Evidence of HBV-DNA integration in bone marrow hematopoietic stem cells (HSC)	8 CHB patients	CD34 + HSC (62.5%)	FISH	Shi et al., 2014

AntiHBc-positive patients were defined as OBI.

PBMCs, Peripheral Blood Mononulear Cells; CHB, chronic hepatitis B; HIV, human immunodeficiency virus; HBsAg, Hepatitis B surface antigen; AHB, Acute Hepatitis B; CI, HBV Chronic Infection; HCC, hepatocellular carcinoma; HSC, Hematopoietic Stem cells; PCR, polymerase chain reaction; FISH, fluorescence in situ hybridization.

More recently, other studies, based on more sensitive and refined molecular assays, have strongly supported the frequent occurrence of HBV-DNA integration, revealing their localization into the genome of PBMCs of HBV chronically infected patients (Laskus et al., 1999; Murakami et al., 2004; Umeda et al., 2005; Wang et al., 2008).

Nevertheless, the pathogenic and clinical implications of HBV lymphotropism and of the related HBV-DNA integrations in lymphoid cells have not yet been fully elucidated. Recently, some studies have supported a potential contribution of HBV-DNA integration in the pathogenesis of immunoproliferative diseases. In particular, HBV has been suggested to contribute to the development of hematological malignancies such as non-Hodgkin Lymphoma (NHL) (Coffin et al., 2021) and more recently it has been revealed that HBV-DNA integration is a common phenomenon in NHL (Li M. et al., 2020). Indeed, the authors identified multiple HBV-DNA integration events in half of the NHL patients analyzed, occurring both in coding and non-coding human regions. Notably, HBV-DNA integration involved the exonic regions (crucial for mRNA synthesis) of four specific genes (FAT2, SETX, ITGA10, and CD63), determining their altered expression and potentially perturbating relevant intracellular pathways. HBV-DNA integration was also found to be recurrent in seven coding genes (ANKS1B, CAPZB, CTNNA3, EGFLAM, FHOD3, HDAC4, and OPCML), which may have potential functions in NHL development. In line with these data, a recent study has shown that HBV-DNA integration profiles in PBMCs are superimposable to those observed in tumor liver tissue, further supporting their role in paving the way toward lymphomagenesis (Lau et al., 2020).

Beyond PBMCs, HBV-DNA integrations have also been detected in HSCs of HBV chronically infected patients (Shi et al., 2014). Such integrations could pave the basis for uncontrolled cell proliferation and in turn for the onset of leukemia.

Overall, further studies are necessary to better elucidate the role of HBV-DNA integration in hematological malignancies.

## Hepatitis B virus integration as a barrier to the achievement of hepatitis B virus cure

The current treatment goal for novel anti-HBV therapies is HBV functional cure, defined as sustained HBsAg loss together with undetectable serum HBV-DNA off-therapy, reflecting the silencing of cccDNA (Lampertico et al., 2017; Lok et al., 2017). Indeed, HBV functional cure represents an optimal therapeutic endpoint, associated with a significantly decreased HCC incidence and no progression to HBV-related cirrhosis (Liu et al., 2016; Yip et al., 2019; Vittal et al., 2022). Unfortunately, this therapeutic goal is rarely achieved by currently available treatment options [nucleos(t)ide analogs (NUCs) and peg-interferon], having a limited effect on cccDNA pool and its activity. However, more recently several new compounds have been developed (immune modulators, capsid assembly modulators, RNA-interference, antisense molecules, entry inhibitors, and HBsAg-release inhibitors), showing greater promise in terms of achieving functional cure.

**FIGURE 4:**
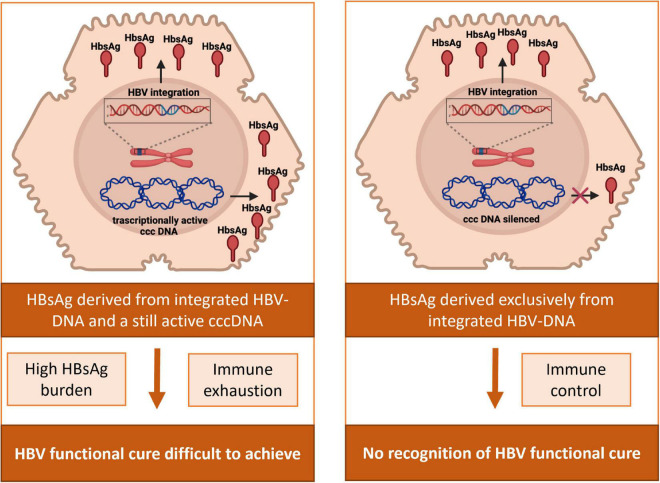
Impact of HBV-DNA integration in the achievement and assessment of HBV functional cure. In patients with an active HBV reservoir, HBsAg can derive from the transcriptional activity of cccDNA and from HBV-integrated DNA. HBsAg from integrated HBV-DNA can consequently contribute to the elevated burden of viral antigens and the subsequent exhaustion of anti-HBV immune response, potentially jeopardizing the achievement of HBV functional cure. In patients with a silenced HBV reservoir, HBsAg in serum can still be present deriving exclusively from integrated HBs-encoding regions, thus hampering the proper recognition of the achievement of HBV functional cure.

In this regard, the occurrence of HBV-DNA integration and the conclusive evidence of HBsAg production derived from integrated HBV-DNA has challenged functional cure, or HBsAg loss, as the ideal therapeutic endpoint (Figure 4). Indeed, the persistence of HBsAg in serum can reflect the continuous production of HBsAg from integrated HBs-encoding regions even despite a completely silenced cccDNA. In keeping with this concept, a recent study estimated that in HBeAg-negative patients, ∼80% of HBsAg transcripts derived from intrahepatic integrated HBV-DNA and did not reflect cccDNA transcriptional activity (Podlaha et al., 2019). Similarly, other studies support a significant contribution of integrated HBV-DNA to serum HBsAg levels in HBeAg-negative patients (Ringlander et al., 2020; Rydell et al., 2020; Meier et al., 2021), as well as in chimpanzees (Wooddell et al., 2017). In these studies, most HBsAg transcripts were characterized by the lack of the conventional HBV polyA signal, typically reflecting transcription of HBs derived from integrated HBV-DNA (Figure 1B).

These findings are in keeping with a study showing that, after 2 years of NUC therapy, HBeAg-negative patients with no evidence of HBV-DNA integration experienced a relevant decay of HBsAg levels, in contrast to those with integrated HBs region, who maintained constant HBsAg levels despite antiviral therapy (HBsAg decline: 2.53 log IU/ml vs. 0.1 log IU/ml, *P* = 0.002) (Hu et al., 2018). Notably, even more minimal change in serum HBsAg levels were observed despite the achievement of undetectable cccDNA in patients receiving long term NUC treatment (Lai et al., 2017).

Overall, these findings support the need for novel biomarkers to better identify patients who have silenced cccDNA, particularly in the light of the multiple novel HBV therapies currently in clinical trials aimed at achieving HBV functional cure.

Furthermore, the production of HBsAg derived from integrated HBV-DNA can contribute to the elevated burden of viral antigens and the subsequent exhaustion of anti-HBV immune response, typically observed in chronic HBV infection (Loggi et al., 2013; Ferrari, 2015; Kim et al., 2020). The role of this phenomenon in jeopardizing the achievement of HBV functional cure merits further investigation (Figure 4).

Beyond functional cure, the ultimate endpoint of anti-HBV treatment is represented by complete sterilizing cure, implying the elimination of both cccDNA and integrated HBV-DNA (Lok et al., 2017; Testoni et al., 2017).

So far, the only approach that has been proposed to achieve this ideal endpoint relies on advanced genome editing strategies such as CRISPR/Cas (clustered regularly interspaced short palindromic repeat/CRISPR associated Cas) (Lin et al., 2014). Although further studies are required, CRISPR/Cas has been shown to be a promising approach to reduce the burden of cccDNA and of integrated HBV-DNA paving the way for a potential sterilizing HBV cure (Wang et al., 2015, 2017; Chen et al., 2021). However, the clinical application of this approach deserves further clarification.

## Overview on different techniques utilized for the analysis of hepatitis B virus integration events

**FIGURE 5:**
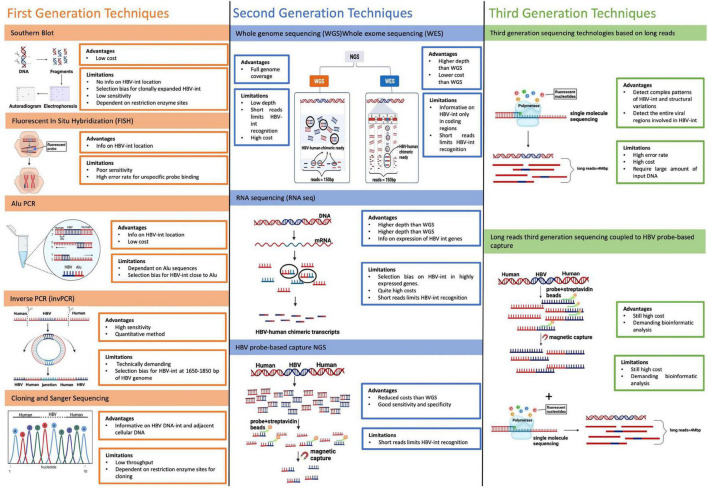
Main techniques utilized to detect HBV-DNA integration. The figure reports an overview of the first-, second-, and third- generation techniques utilized to detect HBV-DNA integration into human genome with their main advantages and limitations. First generation techniques include: Southern Blot, Fluorescent *In situ* Hybridization (FISH), Alu PCR and Inverse PCR (invPCR). Second-generation techniques are based on Next-generation sequencing (NGS) and are based on short reads (with a length of 150 base pair in most used NGS platform). They include: whole-genome sequencing (WGS), whole-exome sequencing (WES), and RNA sequencing (RNA-seq). Third-generation techniques rely on the use of long-read sequencing, a technology based on single-molecule sequencing without pre-PCR amplification, producing long-reads (up to 4 Mbps) in a real-time sequencing process.

Hereinafter, we provide an overview of the first-, second-, and third- generation techniques utilized to detect HBV-DNA integration into human genome with their main advantages and limitations (Figure 5).

### First generation techniques

#### Southern blot

Southern blot was the first technique used for revealing the presence of HBV integration in both tumor tissue and HCC cell lines, in first studies lead in 1980s.

Southern Blot is based on total DNA extraction from HBV-infected cells or tissues, followed by its digestion with a restriction enzyme and a final labeling by 32P-marked HBV hybridization probes. Lastly the digested and labeled DNAs fragments are separated through gel electrophoresis and autoradiographed on films, showing the presence of HBV-DNA integration (Brechot et al., 1980; Chakraborty et al., 1980). This method does not provide any information on the localization of HBV-DNA integration and it suffers a very low sensitivity, since it can detect only HBV integrations present in at least 10^3^–10^5^ copies, biasing HBV integration detection only toward hepatocytes that have undergone clonal expansion (Budzinska et al., 2018a).

#### *In situ* hybridization

*In situ* hybridization (ISH) is a technique detecting HBV integration directly in cells or tissues using a probe, labeled radioactively in first experiments or with a fluorochrome in its more recent evolution [Fluorescent *in situ* hybridization (FISH)]. This technique confers the advantage to identify the location of HBV integration, showing the chromosomal sites of HBV DNA integration (Tokino and Matsubara, 1991; Huang et al., 2005). However, similarly to Southern Blot, ISH is characterized by poor sensitivity and can also be associated with high error rate in HBV integration recognition, due to potential unspecific binding of probes, resulting in a high signal noise, sometimes difficult to interpret.

#### Arthrobacter luteus PCR

Alu-PCR is a modified version of the classical PCR applied for studying HBV integration on the basis of its occurrence close to Alu elements, which are transposable short stretches of DNA recognized by Arthrobacter luteus (Alu) restriction endonuclease, interspersed throughout the entire human genome at a mean interval of about 4 kb (Minami et al., 1995). This technique uses primers pairs, one matching with Alu elements while the other matching with HBV sequence, in order to amplify HBV-human junctions, representing the sites of HBV DNA integrations. Alu-PCR is associated with a higher sensitivity in detecting HBV integration respect to the previously described methods, however, its main bias lies in the possibility to detect only those integrations occurring close to Alu sequences, thus losing the remaining HBV integration events (Murakami et al., 2005; Saitta et al., 2015).

#### Inverse PCR

Inverse PCR (InvPCR) is a refined *ad hoc* designed technique, permitting to amplify the unknown human DNA regions that are adjacent to integrated HBV sequence. InvPCR includes a first step of DNA digestion by using specific restriction enzymes, followed by DNA circularization of cleavage products through self-ligation and amplification by using specific outward HBV-matching primers (Tsuei et al., 1994). This strategy allows to obtain the left, right, or both ends of the HBV-human junction, representing the integrated HBV DNA sequence (Tu and Jilbert, 2017). The major advantage of this method relies in its high sensitivity, permitting to detect HBV integrations, even when occurring as single copy (Tu and Jilbert, 2017). Moreover, it enables to characterize the localization of HBV integration and to quantify their absolute numbers. Unfortunately, invPCR is quite time-consuming and technically demanding, limiting its large use in many laboratories. Moreover, the use of restriction enzymes may limit the detection of some HBV integration junctions due to the lack of the corresponding cutting sites upstream and downstream of the HBV-human DNA junctions (Tu and Jilbert, 2017).

### Second and third generation techniques

The development and refinement of Next-generation sequencing (NGS) methods, characterized by a high sequencing throughput, has given the great opportunity to generate large amount of data on HBV integration occurring in the entire human genome in a high number of samples and in a relatively short time (Ding et al., 2012; Budzinska et al., 2018a).

In NGS-based technologies, the extracted total DNA is randomly fragmented and then amplified and sequenced as millions of short reads (with a length of 150 base pair in most used NGS platform). Afterward, HBV integrations are recognized by specific bioinformatics approaches capable to reveal the presence of HBV-human chimeric reads, containing HBV-human breakpoints, that represents the sites of HBV integration into human genome (Budzinska et al., 2018a; Svicher et al., 2021).

In particular, NGS technologies can be applied for analyzing HBV integrations occurring in the entire human genome by whole-genome sequencing (WGS) or those restricted to human coding regions (exons) by whole-exome sequencing (WES). Furthermore, it is possible to evaluate only the transcriptionally active HBV integrations by RNA sequencing (RNA-seq) (Fujimoto et al., 2012; Jiang et al., 2012; Sung et al., 2012; Shiraishi et al., 2014; Svicher et al., 2021). However, it should be considered that it is crucial to use a deep sequencing coverage in order to achieve a high sensitivity in the detection of HBV integrations by NGS approaches. Unfortunately, this high sequencing coverage necessary to guarantee a high sensitivity in detecting HBV integrations is also associated with high cost. This represents the main disadvantage of these approaches, that constrains their large-scale utilization. Recently, in order to overcome this limitation, a novel NGS approach was developed, based on the preliminary enrichment of HBV-containing sequence fragments by using a set of capture probes, *ad hoc* designed to cover the entire HBV genome. The resulting HBV-enriched sequence library is then sequenced by NGS platform, reducing the necessary sequencing volume to 2 GB per sample and, thus, limiting the relative costs. Overall, HBV probe-based capture technology is more cost-effective while still providing similar specificity and sensitivity to detect viral integration throughout the human genome (Yang et al., 2018; Ishii et al., 2020).

Third-generation techniques to detect HBV-DNA integration rely on the use of long-read sequencing, a technology based on single-molecule sequencing without the pre-PCR amplification step, producing long-reads (up to 4 Mbps) in a real-time sequencing process. This has allowed to overcome the limited length of the DNA reads produced by most NGS platform, to strongly increase the probability to detect HBV integration and, in turn, to analyze extensively the entire HBV regions involved in integration events and the resulting complex interchromosomal genomic rearrangements as fusions and translocations (Alvarez-Benayas et al., 2021; Ramirez et al., 2021; van Buuren et al., 2022). More recently, the long-read sequencing approach has been coupled to the enrichment of HBV sequences by HBV targeting probes, further optimizing the sensitivity of this assay in revealing HBV integrations reducing, at the same time, the costs.

## Conclusion

The availability of more advanced molecular techniques has enabled a deeper understanding of the role of HBV-DNA integration in modulating viral pathogenetic properties. It has been demonstrated that HBV-DNA integration is an early event in HBV infection that can even occur during acute infection and can persist throughout the different phases of chronic infection including occult infection. Integrated HBV-DNA cannot support the production of virions but can represent an important source of viral proteins such as HBsAg. In particular, the sizeable production of HBsAg from HBV-DNA integrants, even in the presence of a transcriptionally silenced cccDNA, has challenged the concept of HBV functional cure, defined as HBsAg-loss, as the ideal therapeutic endpoint. In the light of the multiple novel HBV therapies currently in clinical trials, this has led to much debate in the field about the need for novel biomarkers to better identify patients who have silenced cccDNA.

The events of HBV-DNA integration are particularly abundant during the HBeAg-positive phases of chronic infection, as a consequence of intensive HBV replication and occur with no preferential hotspot in the viral genome. During the process of HBeAg seroconversion, the activation of an efficient anti-HBV immune response can induce a bottleneck, favoring the selection of those hepatocytes in which HBV-DNA integrations have conferred a selective advantage in terms of survival and capability to escape the cytotoxic immune response. This can lead to an enrichment of HBV-DNA integrants in genes involved in the modulation of cell proliferation and apoptosis, promoting the clonal expansion of hepatocytes and representing a first event in mechanisms underlying the neoplastic transformation of the hepatocytes and thus HCC development.

Overall, HBV-DNA integration represents a fascinating and critical element of HBV pathogenetic potential. Moreover, it is emerging as the single most important barrier to achieving both HBV functional cure and sterilizing cure and all novel therapeutic approaches will have to address the effects of HBV-DNA integration to have a meaningful impact on the field. This is an area which will continue to challenge scientists and clinicians and the progress we make will ultimately determine our success in the HBV cure program.

## Author contributions

RS and VS: review conceptualization and writing. SD’A: literature searching, figure preparation, and review writing. LB: literature searching and figure preparation. LP: literature searching. UG: review revision. PK: review conceptualization and revision. All authors contributed to the article and approved the submitted version.
